# Supplementary material for: Probing single electrons across 300-mm spin qubit wafers
Source: Nature. 2024 May 1;629(8010):80–5. doi: 10.1038/s41586-024-07275-6 (PMC11062914; doi:10.1038/s41586-024-07275-6)
Supplement: Supplementary file 1 — Supplementary Sections 1 and 2, including Supplementary Figs 1–3. [file 41586_2024_7275_MOESM1_ESM.pdf]

nature portfolio

https://doi.org/10.1038/s41586-024-07275-6

Supplementary information

Probing single electrons across 300-mm spin qubit wafers

In the format provided by the authors and unedited

Supplementary Information: Probing single electrons across 300 mm spin qubit wafers

Samuel Neyens*,†, Otto Zietz*, Thomas F. Watson, Florian Luthi, Aditi Nethwewala, Hubert George, Eric Henry, Mohammad Islam, Andrew Wagner, Felix Borjans, Elliot J. Connors, J. Corrigan, Matthew J. Curry, Daniel Keith, Roza Kotlyar, Lester F. Lampert, Mateusz T. Mądzik, Kent Millard, Fahd A. Mohiyaddin, Stefano Pellerano, Ravi Pillarisetty, Mick Ramsey, Rostyslav Savytskyy, Simon Schaal, Guoji Zheng, Joshua Ziegler, Nathan Bishop, Stephanie Bojarski, Jeanette Roberts, and James S. Clarke†

Intel Corp., 2501 NE Century Blvd, Hillsboro, OR 97124, USA

**These authors contributed equally to this work*

†*Corresponding authors: samuel.neyens@intel.com; james.s.clarke@intel.com*

I. SUPERCONDUCTING LAYER CHARACTERIZATION

To further characterize the superconducting metal layers used to reduce gate line resistance, we perform measurements in a conventional cryostat with magnetic field control (Quantum Design PPMS Dynacool). These measurements are taken at a temperature of 1.7 K, below the critical temperatures of both layers, which are found to be ∼2.4 K and ∼6.5 K for the gate and interconnect layer, respectively. Supplementary Fig. 1a-d shows measurements of critical current (I_C) and in-plane critical magnetic field ($H_{C,||}$) for both the superconducting interconnect and superconducting gate layers. All of these measurements are taken in a four-point resistance configuration. We find I_C of 300 μA and 8 μA for the superconducting interconnect and gate layer, respectively. The IV sweeps also show a strong hysteresis effect in the superconducting gate layer (Supplementary Fig. 1b). We measure $H_{C,||}$ of 2.7 T and 0.7 T for the superconducting interconnect and gate layer, respectively. In the magnetic field sweep for the superconducting gate layer (Supplementary Fig. 1d), we observe two resistance transitions, at 0.7 T and 2.6 T, respectively, so we report the transition at lower field to be $H_{C,||}$.

While both layers have critical fields above the typical operating point of spin qubits, the superconducting gate layer has a much lower critical current and greater hysteresis compared to the interconnect layer, which together may limit these gates' usefulness for applying microwave power. We also note that superconducting gates may not be suitable for all spin qubit applications, particularly if the Meissner effect contributes a significant magnetic field gradient,

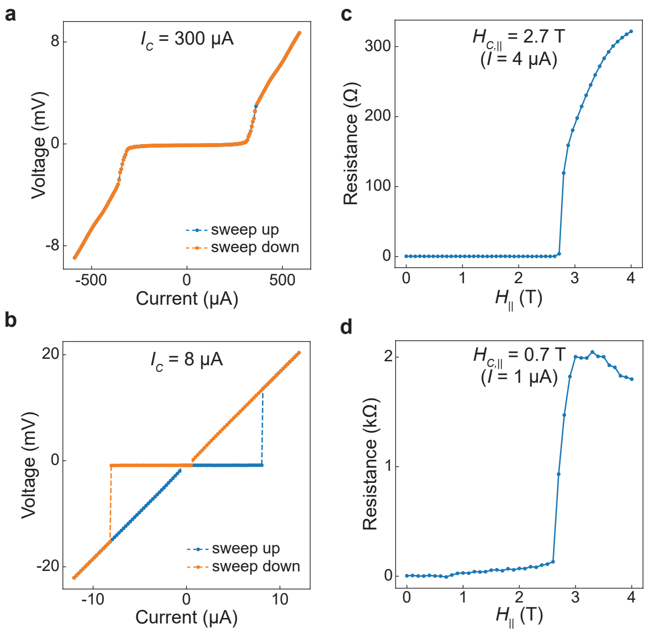

Supplementary Figure 1. **Characterization of superconducting metal layers in a spin qubit device process. a-b**, IV sweeps to measure critical current (I_C), showing measured voltage as a function of applied current, for test structures with superconducting interconnect (**a**) and gate (**b**) layers. **c-d**, Measurements of critical in-plane magnetic field ($H_{C,||}$), showing resistance as a function of in-plane magnetic field, for test structures with superconducting interconnect (**c**) and gate (**d**) layers.

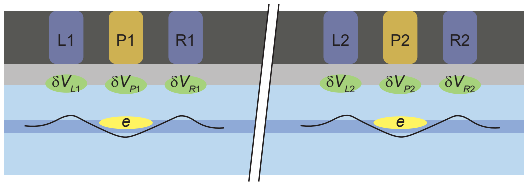

Supplementary Figure 2. **Cartoon of matched pair 1e voltage measurement configuration.** In this configuration, 1e voltage is compared between mirror-symmetric quantum dot pairs, each with one plunger and two barrier gates. Every gate is taken to have a voltage offset (δV_i) reflecting the adjustment needed to tune the chemical potential under that gate in response to local variation.

which can decrease the fidelity of exchange-only qubits [1]. In the case of our devices, the interconnect layer is far enough from the qubits that induced Meissner-effect fields from this layer should be negligible. Furthermore, since the interconnect layer contributes significantly more capacitance than the gates themselves, using only a superconducting interconnect layer may be sufficient to reduce on-chip heating during microwave driving and/or the application of fast pulses.

II. ASSESSING THE IMPACT OF BARRIER VOLTAGE TUNING ON 1e PLUNGER VOLTAGE VARIATION

Here we estimate the impact of fine-tuning the barrier gates on the resulting variation in 1e plunger voltage. We consider the case where each quantum dot is defined by a plunger gate and two barrier gates. Supplementary Fig. 2 shows a cartoon of the approximate configuration including two mirror-symmetric quantum dots 1 and 2 defined by plunger gate P1(2), left barrier gate L1(2), and right barrier gate R1(2). First we consider the relationship between the voltages needed to tune a single quantum dot. In the absence of any cross-capacitance, the plunger voltage needed to tune the electron number to 1e can be written as $V_P = V_{0,P} + \delta V_P$, where $V_{0,P}$ is the theoretical 1e voltage for the gate in the absence of any disorder and δV_P is the voltage offset needed to tune to 1e in the presence of disorder. Here δV_P represents how much the gate voltage must change in order to tune to a target chemical potential and can be thought of as a gate voltage-referred metric of local variation. Similarly, if the barrier gates are fine-tuned to target a specific tunnel rate, the voltage of a left (right) barrier can be written as $V_{L(R)} = V_{0,L(R)} + \delta V_{L(R)}$, where $V_{0,L(R)}$ is the theoretical setpoint in the absence of disorder and $\delta V_{L(R)}$ is the voltage offset needed to tune the tunnel rate in the presence of local disorder. When cross-capacitance is introduced, all voltage setpoints become reduced by the presence of surrounding voltages, resulting in a set of three coupled equations for gate voltages:

$$V_P = V_{0,P} + \delta V_P - c_1 V_L - c_1 V_R, \tag{1}$$

$$V_L = V_{0,L} + \delta V_L - c_1 V_P - c_2 V_R, \tag{2}$$

$$V_R = V_{0,R} + \delta V_R - c_1 V_P - c_2 V_L, \tag{3}$$

where $c_{1(2)}$ is the relative cross-capacitance between gates separated by 1(2) gate pitches. Solving these equations leads to a new formula for the plunger voltage needed to tune to 1e:

$$V_P = \frac{(1+c_2)V_{0,P} - c_1 V_{0,L} - c_1 V_{0,R} + (1+c_2)\delta V_P - c_1 \delta V_L - c_1 \delta V_R}{1 + c_2 - 2c_1^2}. \tag{4}$$

As this formula shows, cross-capacitance to the neighboring barrier gates causes the plunger voltage to become coupled to disorder local to the barrier gates, because fine-tuning those barrier voltages in response to their environment introduces corrections to the voltage needed to tune the plunger. For the case where barrier gates are held at a fixed voltage and not fine-tuned to compensate for local disorder, this disorder does not couple in to the plunger voltage, and the analogous formula can be obtained by setting δV_L and δV_R to 0.

The variance of the plunger voltage can then be expressed in terms of the variances of the three voltage offsets:

$$\begin{aligned}\sigma_{V_P}^2 = \big((1+c_2)^2\sigma_{\delta V_P}^2 + c_1^2\sigma_{\delta V_L}^2 + c_1^2\sigma_{\delta V_R}^2 - 2c_1(1+c_2)\sigma_{\delta V_P}\sigma_{\delta V_L}\rho_{PL} \\ - 2c_1(1+c_2)\sigma_{\delta V_P}\sigma_{\delta V_R}\rho_{PR} + 2c_1^2\sigma_{\delta V_L}\sigma_{\delta V_R}\rho_{LR}\big)(1+c_2-2c_1^2)^{-2},\end{aligned} \tag{5}$$

where ρ_{ij} is the correlation coefficient between voltage offset variables δV_i and δV_j. This expression can be simplified by assuming that all voltage offsets have the same variance and that the correlation coefficients between all pair of gates within a device are the same, giving:

$$\sigma_{V_P}^2 = \frac{(1+c_2)^2 + 2c_1^2 - 4c_1(1+c_2-c_1/2)\rho}{(1+c_2-2c_1^2)^2}\sigma_{\delta V}^2, \tag{6}$$

where $\sigma_{\delta V}^2$ is the variance of each voltage offset. A similar variance can be obtained for the case where barriers are fixed, and taking the ratio between the two reveals the relative impact of tuning the barriers on the variance:

$$\frac{\sigma_{V_P,\text{tuned}}^2}{\sigma_{V_P,\text{fixed}}^2} = 1 + \frac{2c_1^2 - 4c_1(1+c_2-c_1/2)\rho}{(1+c_2)^2}. \tag{7}$$

This expression shows that the effect of tuning the barrier voltages can be either to increase or to decrease the variance of V_P, depending on the cross-capacitances involved and the correlation between voltage offsets within a device. Roughly speaking: with high correlation between voltage offsets, tuning the barrier voltages can "help" the tuning of the plunger voltage, decreasing $\sigma_{V_P}^2$; while with low correlation between voltage offsets, tuning the barrier voltages can "hurt" the tuning of the plunger voltage, increasing $\sigma_{V_P}^2$.

Next we consider the matched pair analysis where we report the standard deviation of the difference between pairs of $1e$ voltages within a device. In the simple transistor case, this method subtracts the effects of correlations in order to obtain the variation that is uncorrelated on the length scale separating the gate pairs. The variance of the difference between two such voltages can be written as

$$\frac{\sigma_{\Delta V}^2}{2} = (1-\rho)\sigma_V^2, \tag{8}$$

where σ_V^2 is the variance of the individual gate voltages and ρ is the correlation coefficient between the two voltage distributions. To assess the impact of tuning the barrier voltages on the variance of $1e$ voltage differences, we consider the two plunger gates P1 and P2 from Supplementary Fig. 2 and take the difference between their voltages (using Eq. 4):

$$\Delta V_P = \frac{(1+c_2)\delta V_{P1} - c_1\delta V_{L1} - c_1\delta V_{R1} - (1+c_2)\delta V_{P2} + c_1\delta V_{L2} + c_1\delta V_{R1}}{(1+c_2-2c_1^2)}, \tag{9}$$

where the fixed reference voltages $V_{0,i}$ are assumed to be symmetric between the two quantum dots and therefore cancel out. This quantity is a function of six voltage offsets; taking the variance of this quantity yields a lengthy expression with six diagonal terms and fifteen cross terms proportional to all the correlation coefficients between gate pairs. Similarly to the case of $\sigma_{V_P}^2$, the formula for $\sigma_{\Delta V_P}^2$ can be simplified by assuming that all voltage offsets have the same variance and that the correlation coefficients between all pair of gates within a device are the same, yielding:

$$\frac{\sigma_{\Delta V_P}^2}{2} = \frac{2c_1^2 + (1+c_2)^2}{(1+c_2-2c_1^2)^2}(1-\rho)\sigma_{\delta V}^2. \tag{10}$$

Again an analogous expression can be generated for the case of fixed barriers by setting the barrier voltage offset variables to zero, and the relative impact of tuning the barriers can be estimated by the ratio:

$$\frac{\sigma_{\Delta V_P,\text{tuned}}^2}{\sigma_{\Delta V_P,\text{fixed}}^2} = 1 + \frac{2c_1^2}{(1+c_2)^2}. \tag{11}$$

This expression shows that for the matched pair analysis method, the effect of tuning the barriers is strictly to increase the variance. This can be interpreted as a result of the matched pair analysis subtracting out the impact of device-level voltage correlations, while the uncorrelated component of barrier voltage offsets that couples to the plunger through cross-capacitance still adds in quadrature. Of course, both $\sigma_{\Delta V_P,\text{tuned}}^2$ and $\sigma_{\Delta V_P,\text{fixed}}^2$ will decrease with increasing ρ, but their ratio is strictly ≥ 1 by this estimation.

To test this effect, we collect two sets of charge sensing datasets on a new wafer with a 50 nm SiGe barrier. For the first set of devices, we collect V_{1e} using our standard method of tuning the barrier voltages, and for the second set of devices, we collect V_{1e} with barrier gates set to a fixed value of 0.65 V. The resulting V_{1e} and matched pair ΔV_{1e} distributions are shown in Supplementary Fig. 3a-d. Similar to the analytical findings, we observe that fine-tuning the barrier voltages has the effect of decreasing the standard deviation of the V_{1e} distribution while increasing the

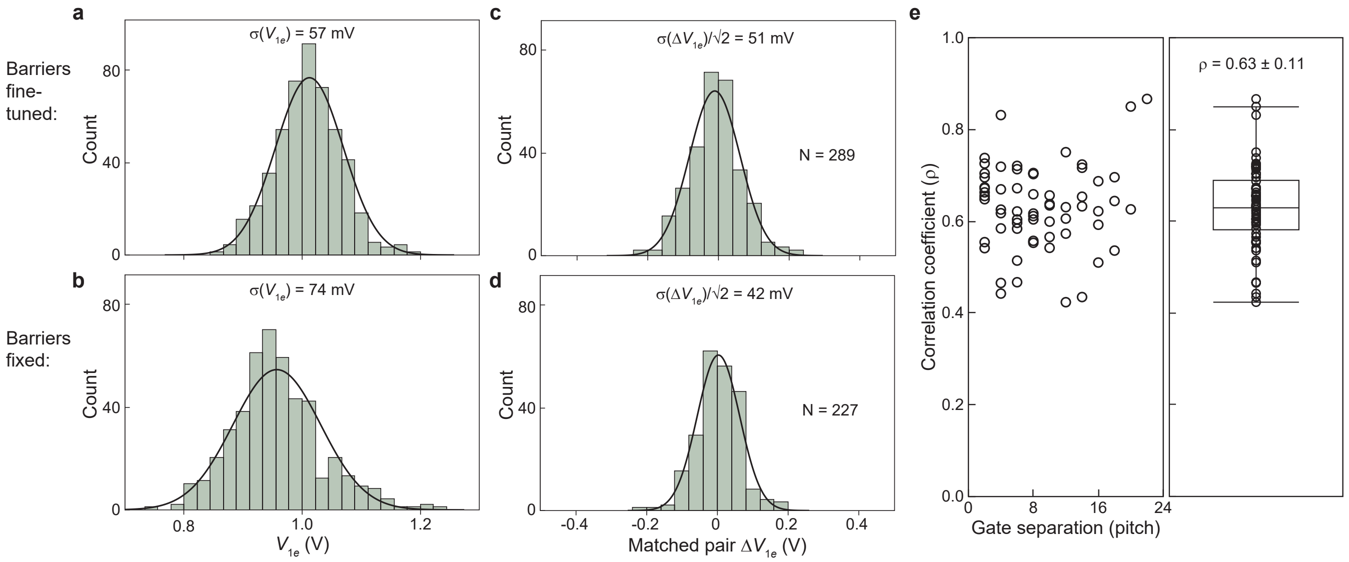

Supplementary Figure 3. **Impact of barrier voltage tuning on matched pair 1e voltage analysis.** 1e voltage (V_{1e}) datasets collected on a wafer with 50 nm SiGe barrier (a different wafer from those analyzed in the main text). Histograms showing metrics of V_{1e} variation are shown after two methods of data collection: using our standard method of fine-tuning the barrier voltages from barrier-barrier scans and using an alternate method where barrier voltages are set to a fixed value of 0.65 V. **a-b**, Raw V_{1e} distributions taken with barriers fine-tuned (**a**) and barriers fixed (**b**). The plungers on the edge of the array (P1 and P12) are excluded due to their proximity to the accumulation gates resulting in systematic shifts to their V_{1e} median. **c-d**, Matched pair ΔV_{1e} distributions taken with barriers fine-tuned (**c**) and barriers fixed (**d**). **e**, Correlation coefficients (ρ) for V_{1e} data calculated between the 66 unique gate pairs in the 12 quantum dot array. Correlations are calculated from the V_{1e} data collected with barriers fixed. The left panel shows correlation coefficient as a function of the separation distance between gate pairs in units of gate pitch (60 nm). The right panel summarizes all coefficients in one scatter plot, giving a median value of $\rho = 0.63 \pm 0.11$. Box plots display the median and inter-quartile range (IQR) of each distribution. Whiskers mark the maximum and minimum values excluding outliers, which are defined as points removed from the median by more than 1.5 times the IQR.

standard deviation of the matched pair ΔV_{1e} distribution. In the former case, this is due to the presence of device-level correlations between gates, while in the latter case, this is due to the matched pair analysis method factoring out device-level correlations while preserving the impact of uncorrelated voltage offsets from the barrier gates. To assess the magnitude of device-level correlation present between gates, we also calculate correlation coefficients (ρ) between all 66 unique gate pairs in the 12-quantum-dot array using the V_{1e} dataset collected with fixed barrier voltages, yielding a median value of $\rho = 0.63 \pm 0.11$ (see Supplementary Fig. 3). We also observe no systematic dependence of correlation coefficient on the separation distance between gate pairs within the array, confirming that the approximation of all gate pairs having the same device-level correlation coefficient is reasonable.

As a quantitative check, we take typical values of nearest- and next-nearest-neighbor cross-capacitance from these devices with 50 nm SiGe barrier ($c_1 = 0.55 \pm 0.05$ and $c_1 = 0.15 \pm 0.05$) and plug them into Eq. 7 and Eq. 11, along with the extracted value of ρ. Taking the square root of these expressions gives the expected ratios for the standard deviations of V_{1e} and ΔV_{1e} to be 0.7 ± 0.2 and 1.2 ± 0.1, respectively, in agreement with the measured ratios of 0.77 and 1.2. This confirms the analytical conclusion that tuning the barrier voltages does not result in under-reporting of variation when the standard deviation of matched pair ΔV_{1e} is used.

[1] A. J. Weinstein, M. D. Reed, A. M. Jones, R. W. Andrews, D. Barnes, J. Z. Blumoff, L. E. Euliss, K. Eng, B. H. Fong, S. D. Ha, D. R. Hulbert, C. A. C. Jackson, M. Jura, T. E. Keating, J. Kerckhoff, A. A. Kiselev, J. Matten, G. Sabbir, A. Smith, J. Wright, M. T. Rakher, T. D. Ladd, and M. G. Borselli, Universal logic with encoded spin qubits in silicon, Nature **615**, 817 (2023).
